# Cocktail of *Astragalus Membranaceus* and *Radix Trichosanthis* Suppresses Melanoma Tumor Growth and Cell Migration Through Regulation of Akt-Related Signaling Pathway

**DOI:** 10.3389/fphar.2022.880215

**Published:** 2022-06-01

**Authors:** Qiuyan Zhang, Lei Gao, Songli Huang, Yuxi Liang, Jingyan Hu, Yuan Zhang, Shengli Wei, Xiuhua Hu

**Affiliations:** ^1^ School of Life Sciences, Beijing University of Chinese Medicine, Beijing, China; ^2^ Dongfang Hospital, Beijing University of Chinese Medicine, Beijing, China; ^3^ School of Traditional Chinese Medicine, Beijing University of Chinese Medicine, Beijing, China; ^4^ School of Chinese Materia Medica, Beijing University of Chinese Medicine, Beijing, China

**Keywords:** Astragalus Membranaceus, Radix Trichosanthis, herbal “cocktail”, Akt-related singnaling pathway, proliferation, migration

## Abstract

**Background:** Malignant melanoma has high morbidity and mortality and limited treatment options. Traditional Chinese medicine has great potential in the clinical therapy of cancer, and the theory of compatibility is one core content of Chinese medical theory. *Astragalus Membranaceus* and *Radix Trichosanthis* are clinically effective for the treatment of various cancers.

**Methods:** We verified the effects of AMD, RTD, and their “cocktail” on melanoma model *in vitro* and *in vivo* and the mechanism of its effect on the Akt-related signaling pathway by network pharmacology, MTT, flow cytometry, LDH, SOD, MDA assay, and Western blot.

**Results:** The network pharmacology analysis indicated that the PI3K-Akt pathway plays a crucial role in the treatment of malignant melanoma with these two herbs. In addition, AMD, RTD, and their “cocktail” could inhibit the proliferation of A375 cells by reducing the survival rate in a concentration-dependent manner and by regulating the cell cycle, and the compatibility of two herbs also could inhibit melanoma growth. They could, respectively, induce apoptosis and inhibit migration by affecting the expression of Bcl-2, Bax, p53, snail, E-cadherin, and N-cadherin. Furthermore, LDH activity was decreased, while SOD increased and MDA reduced. The factors of the Akt-related signaling pathway, Akt and p-Akt, were decreased.

**Conclusion:** This study showed that AMD, RTD, and their “cocktail” could regulate cell proliferation, apoptosis, and metastasis in A375 cells through the suppression of the Akt-related signaling pathway, and the “cocktail” groups had detoxification and additive effects. The best compatibility of the two herbs also can inhibit tumor growth and metastasis *in vivo.*

## Introduction

Malignant melanoma (MM) is a malignant cancer characterized by increasing incidence and high metastatic capacity and which is originated from the dysplasia of melanocytes. In America, MM ranks third as the most common cancer in men and fifth in women (Miller et al., 20192019). Although the overall cancer incidence rate is reportedly in decline, the incidence of MM continues to increase, with an estimated annual increase of approximately 3%. In addition, the patients with MM are mostly younger, with 47% of the patients ≤65 years old and 33% ≤ 50 years (Miller et al., 20192019). Surgery is the main treatment for early-stage MM, but it has a frequent postoperative recurrence due to early malignant invasion. Chemotherapy and radiotherapy are employed in patients unsuitable for surgery and patients refusing surgery, but they are prone to side effects and poor prognosis (Davis et al., 2019). Therefore, there is an urgent need to seek new treatment methods for MM. Moreover, the potential values of traditional Chinese medicine (TCM) in the treatment of melanoma have gradually been revealed.

*Astragalus Membranaceus* (AM) and *Radix Trichosanthis* (RT) are two herbs widely used in TCM to treat different cancers, which have been proven to inhibit tumor growth and metastasis (Zhu et al., 2021; Bian et al., 2022). Chinese herbal compound formula is used most commonly in the clinic, and it is more effective when certain specific herbs and certain ratios are used together. In ancient TCM books, such as “Yixue Zhongzhong Canxilu” written by Zhang Xichun in the Qing dynasty and “Orthodox Manual of External Medicine” written by Chen Shigong in the Ming dynasty, there are also cases of compatibility of AM and RT for disease, especially diabetes. The compatibility of TCM is like mixing a cocktail, so we called it “cocktail” here. Therefore, it has the positive significance of clinical treatment to research the effect of AM, RT, and their “cocktail” on MM cells. However, the specific mechanism of AM and RT in MM treatment is still unclear. In this research, we performed a network pharmacological analysis and evaluated the influence on the tumor growth and proliferation, apoptosis, and migration of MM cells with different concentrations of AM, RT, and their “cocktail,” which might provide a reference to clinical applications. We also researched the influence on factors related to apoptosis and migration; the change of LDH activity, SOD activity, and MDA content; and the effect on the Akt-related signaling pathway, which might reflect the mechanisms of the treatment of AM, RT, and their “cocktail.”

## Materials and Methods

### Herb Preparation

Every 100 g herb (AM and RT, from Beijing Tongrentang) was soaked in deionized water for 1 h and decocted twice, separately. The decoction was poured into a beaker for 50 ml concentration, transferred, and centrifuged at 1000 *g* for 20 min. The supernatants, respectively, were the *Astragalus Membranaceus* decoction (AMD) and *Radix Trichosanthis* decoction (RTD).

### LC-ESI-MS/MS

The LC-ESI-MS/MS analysis system was equipped with an ultrahigh-performance liquid chromatograph (UHPLC) and an MS system. A Waters ACQUITY UPLC HSS T3 C18 column (2.1 mm × 100 mm, 1.8 μm) was used at 30°C and 5 ul of injection volume. Gradient elution conditions were set as given in Supplementary Table S1. MS parameters were a sheath gas flow of 40 arb, an auxiliary gas flow rate of 15 arb, a capillary temperature of 320°C, an auxiliary gas heater temperature of 350°C, and a positive spray voltage of 3.2 kv. The resolution of MS and MS/MS was 70,000 and 17,500, respectively.

### Network Pharmacology

The research process is shown in Supplementary Figure S1. The active chemical compositions of AM and RT were retrieved from the Traditional Chinese Medicine System Pharmacology (TCMSP) database and screened, which conformed to the oral bioavailability (OB) ≥ 30% and drug-likeness (D)L ≥ 0.18. The potential targets of herbs were further screened in target relative databases and completely reserved. The known potential melanoma-related targets were found with “*Homo sapiens*” in disease target databases and merged and deleted replicas of all targets. We used the STRING database to establish a protein–protein interaction (PPI) network and visualized the PPI by Cytoscape software. Subsequently, KEGG analysis was performed by linking targets to the Database for Annotation, Visualization, and Integrated Discovery database (DAVID). The drug–target–pathway–disease network was established via Cytoscape software.

### MTT Assay

Human malignant melanoma A375 cells and human L-02 liver cells (purchased from Cell Resource Center, Peking Union Medical College) were plated onto 96-well plates with 3×10^3^ cells/well. A control group (medium), AMD groups (medium with concentrations 2, 4, 8, 10, and 20 mg/ml of AMD), and RTD groups (medium with concentrations 0.16, 0.32, 0.64, 1.28, and 2.56 mg/ml of RTD) were set and incubated for 48 h or 72 h. After MTT solution and DMSO were used successively, the OD value was estimated at an absorbance of 490 nm. IC_0_, IC_25_, and IC_50_ (the cell inhibitory rates were 0, 25, and 50%, respectively) were calculated. Afterward, the control group, different concentrations of AMD, RTD, and “cocktail” groups were set up for further tests.

### Animal Experiments

Male and female C57BL/6J mice aged 6–8 weeks (purchased from Huafukang Biotechnology) were randomly divided into six groups of six with the abdomen depilated. Following the experimental progress (Figure 4A), 0.1 ml PBS was subcutaneously injected at the abdomen of the mice of the normal control group (NCG), while suspension (prepared by 0.1 ml of PBS) with 5 × 10^5^ B16-F10 cells were injected into others. This day was marked as d0. On d2, small black spots greater than 2 mm diameter appeared at the injection site. At d3, the NCG and model control group (MCG) were given ddH_2_O intragastric administration, while the three compatible groups were given AMD and RTD solution at a ratio of 5:3 (L 3.12 g/kg, M 6.24 g/kg, and H 12.48 g/kg, respectively), and the positive control group (PCG) was given compound cyclophosphamide solution (19.5 mg/kg) once a day for 14 consecutive days. The mice weight, tumor length, and transverse diameter were recorded every second day, and the tumors of MCG were photographed every 4 days. Finally, the mice were anesthetized, and the blood, tumor, and liver were collected. The pathological features were determined by H&E staining. All these experiments were in accordance with the guidelines of the Animal Care and Use Committee.

### Flow Cytometry Assay and Hoechst Staining

After treating A375 cells with AMD, RTD, and their “cocktail” groups, the cell cycle and apoptosis (processed by an Annexin-FITC Apoptosis Detection Kit) were observed by flow cytometry. In addition, we stained the cells with Hoechst 33342 and photographed them under a confocal microscope (400x image) to observe the apoptotic morphology.

### Scratch Wound Healing Assay

After A375 cells were cultured for 24 h, a cross was scraped at the center of wells by using a 10-µl micropipette tip and photographed to record. Then, the cells were cultured after they were treated with different concentrations of AMD, RTD, and their “cocktail.” After 48-h or 72-h treatment, images were captured again.

### LDH, SOD, and MDA Assay

The treatment of A375 cells was consistent as mentioned before; then, the cell supernatant (treated for 48 h) and mice serum were collected (treated for 14 days). As per the guidelines of the corresponding kit manufacturer’s instructions, the value of LDH, SOD, and MDA was measured. Finally, the results were calculated by using the formula given in the instructions.

### Western Blot Assay

The protein in tissues and cells were extracted to determine protein concentration by using a BCA protein detection kit. Western blot experiments for *β*-actin, Bcl-2, Bax, p53, E-cadherin, N-cadherin, snail, Akt, and p-Akt were performed and photographed by using the Gel imaging instrument. ImageJ software was used to analyze the bands.

### Statistical Analysis

The experiments were repeated at least three times, and differences between all data were evaluated using ANOVA, and *p* < 0.05 was considered statistically significant.

## Results

### Analysis of Chemical Components in AMD, RTD, and Their Cocktail

**FIGURE 1:**
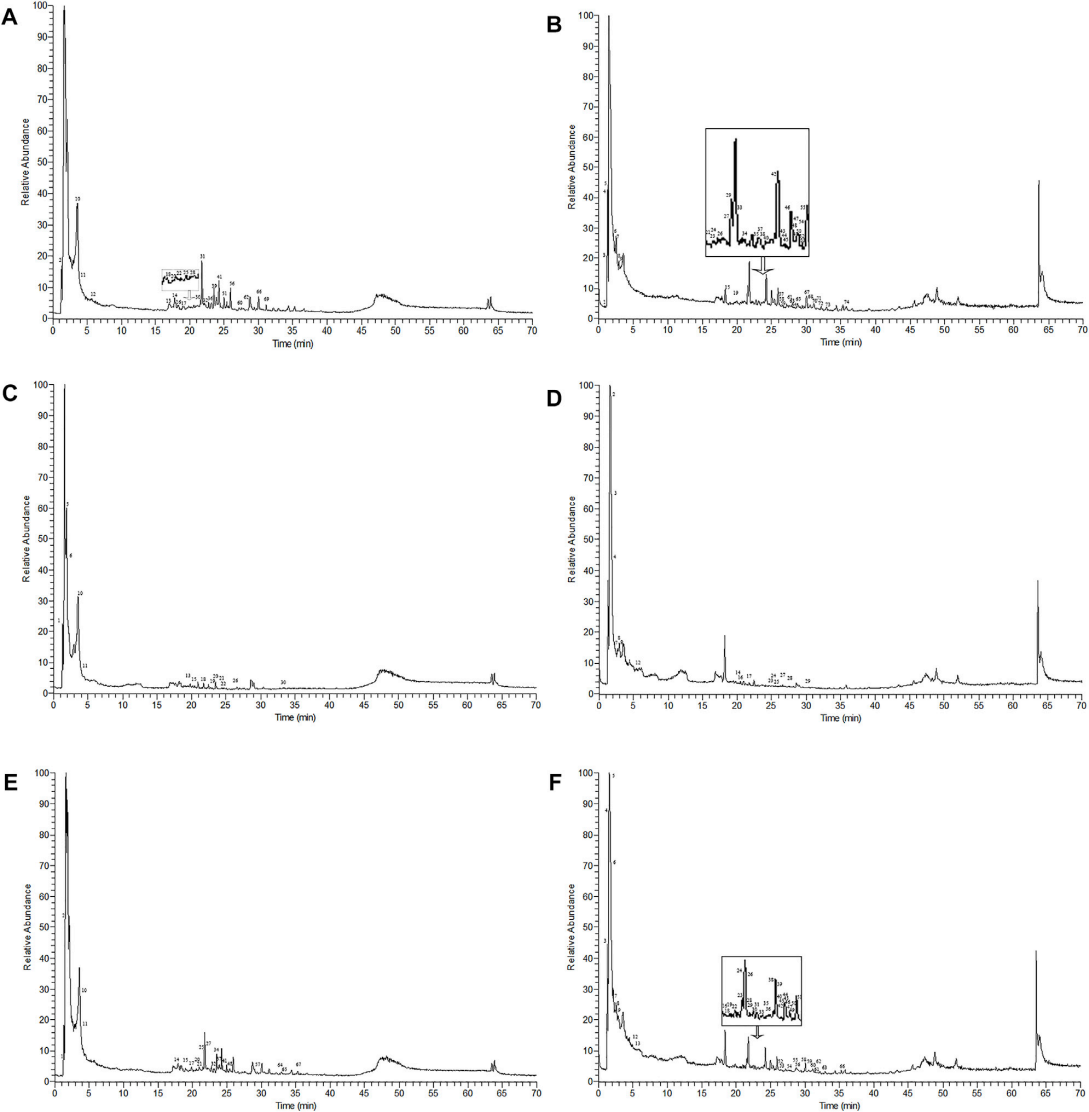
Mass spectra of AMD, RTD, and compatibility of AMD and RTD in negative and positive ion modes. **(A) **Mass spectra of AMD in the negative ion mode. **(B)** Mass spectra of AMD in the positive ion mode. **(C)** Mass spectra of AMD in the negative ion mode. **(D)** Mass spectra of RTD solutions in the positive ion mode. **(E)** Mass spectra of the compatibility of AMD and RTD with a ratio of 5:3 in the negative ion mode. **(F)** Mass spectra of the compatibility of AMD and RTD with a ratio of 5:3 in the positive ion mode.

The Compound Discoverer 3.1.0.305, mzCloud, and mzVault databases were used to identify unknown compounds. The chemical components are listed in Supplementary Table S2, S3 and Table 1. There were 25 and 49 chemical components of AMD and 14 and 16 components of RTD in the negative and positive ion modes, respectively (Figure 1), while 20 and 47 components were observed in their cocktail at a ratio of 5:3 in different ion modes (Table 1). The AMD compounds include flavonoids, organic acids, and amino acids. Terpenoids, organic acids, and amino acids are the main compounds in RTD. Compounds of the cocktail of AMD and RTD are also flavonoids, organic acids, and amino acids.Interestingly, cocktail contains some unique compounds like berberine and diosmetin-7-O-β- Dglucopyranoside.

**TABLE 1:** Information of compounds of compatibility of AMD and RTD with a ratio of 5 to 3 detected by LC-ESI-MS/MS in different Ion modes.

No.	RT [min]	Ion mode	Name	Formula
1	1.292	[M-H]^−1^	3-[(Carboxycarbonyl)amino]-l-alanine	C_5_ H_8_ N_2_ O_5_
2	1.551	[M-H]^−1^	l-Glutamic acid	C_5_ H_9_ N O_4_
3	1.555	[M + H]^+1^	l-Threonine	C_4_ H_9_ N O_3_
4	1.685	[M + H]^+1^	Trigonelline HCl	C_7_ H_7_ N O_2_
5	1.7	[M + H]^+1^	2-Pyrrolidinecarboxylic acid	C_5_ H_9_ N O_2_
6	1.718	[M + H]^+1^	Betaine	C_5_ H_11_ N O_2_
7	2.428	[M + H]^+1^	l-Valine	C_5_ H_11_ N O_2_
8	2.913	[M + H]^+1^	Adenine	C_5_ H_5_ N_5_
9	2.952	[M + H]^+1^	Nicotinic acid	C_6_ H_5_ N O_2_
10	3.574	[M-H]^−1^	Citric acid	C_6_ H_8_ O_7_
11	4.03	[M-H]^−1^	Maleic acid	C_4_ H_4_ O_4_
12	5.367	[M + H]^+1^	l-Tyrosine	C_9_ H_11_ N O_3_
13	5.404	[M + H]^+1^	p-Coumaric acid	C_9_ H_8_ O_3_
14	17.872	[M-H]^−1^	Asperulosidic acid	C_18_ H_24_ O_12_
15	19.059	[M-H]^−1^	4-Hydroxybenzoic acid	C_7_ H_6_ O_3_
16	20.156	[M + H]^+1^	Asarylaldehyde	C_10_ H_12_ O_4_
17	20.228	[M-H]^−1^	Sibiricose A5	C_22_ H_30_ O_14_
18	20.257	[M + H]^+1^	l-Phenylalanine	C_9_ H_11_ N O_2_
19	20.557	[M + H]^+1^	Abscisic acid	C_15_ H_20_ O_4_
20	20.712	[M-H]^−1^	Diosmetin-7-O-β-d-glucopyranoside	C_22_ H_22_ O_11_
21	20.894	[M-H]^−1^	Tenuifoliside A	C_31_ H_38_ O_17_
22	20.942	[M + H]^+1^	7-Methoxycoumarin	C_10_ H_8_ O_3_
23	21.647	[M + H]^+1^	2-Hydroxy-4-methoxybenzaldehyde	C_8_ H_8_ O_3_
24	21.741	[M + H]^+1^	Calycosin-7-O-β-D-glucoside	C_22_ H_22_ O_10_
25	21.773	[M-H]^−1^	L-Tryptophan	C_11_ H_12_ N_2_ O_2_
26	22.015	[M + H]^+1^	Liquiritigenin	C_15_ H_12_ O_4_
27	22.016	[M-H]^−1^	Liquiritin	C_21_ H_22_ O_9_
28	22.076	[M + H]^+1^	3,5-Dimethoxy-4-hydroxybenzaldehyde	C_9_ H_10_ O_4_
29	22.468	[M + H]^+1^	Genistin	C_21_ H_20_ O_10_
30	22.726	[M + H]^+1^	Tectoridin	C_22_ H_22_ O_11_
31	22.878	[M + H]^+1^	Artemisinin	C_15_ H_22_ O_5_
32	23.187	[M-H]^−1^	Phloridzin	C_21_ H_24_ O_10_
33	23.194	[M + H]^+1^	Isosakuranetin	C_16_ H_14_ O_5_
34	23.522	[M-H]^−1^	Azelaic acid	C_9_ H_16_ O_4_
35	23.541	[M + H]^+1^	Jatrorrhizine	C_20_ H_19_ N O_4_
36	23.66	[M + H]^+1^	Epiberberine	C_20_ H_17_ N O_4_
37	23.898	[M-H]^−1^	Salicylic acid	C_7_ H_6_ O_3_
38	24.055	[M + H]^+1^	6″-O-Acetylglycitin	C_24_ H_24_ O_11_
39	24.219	[M + H]^+1^	Baicalin	C_21_ H_18_ O_11_
40	24.243	[M + H]^+1^	Ononin	C_22_ H_22_ O_9_
41	24.45	[M-H]^−1^	Hydroxygenkwanin	C_16_ H_12_ O_6_
42	24.534	[M + H]^+1^	Ligustilide	C_12_ H_14_ O_2_
43	24.98	[M + H]^+1^	Palmatine	C_21_ H_21_ N O_4_
44	25.005	[M + H]^+1^	Methylnissolin-3-O-glucoside	C_23_ H_26_ O_10_
45	25.011	[M + H]^+1^	4′,7-Di-O-methylnaringenin	C_17_ H_16_ O_5_
46	25.013	[M + H]^+1^	o-Veratraldehyde	C_9_ H_10_ O_3_
47	25.239	[M + H]^+1^	Berberine	C_20_ H_17_ N O_4_
48	25.383	[M-H]^−1^	Isomucronulatol 7-O-glucoside	C_23_ H_28_ O_10_
49	25.385	[M + H]^+1^	Benzoic acid	C_7_ H_6_ O_2_
50	25.933	[M + H]^+1^	Calycosin	C_16_ H_12_ O_5_
51	26.178	[M + H]^+1^	Wogonoside	C_22_ H_20_ O_11_
52	26.329	[M + H]^+1^	Pectolinarigenin	C_17_ H_14_ O_6_
53	26.448	[M + H]^+1^	Glycitin	C_22_ H_22_ O_10_
54	27.973	[M + H]^+1^	Artemisinic acid	C_15_ H_22_ O_2_
55	28.624	[M + H]^+1^	Resibufogenin	C_24_ H_32_ O_4_
56	28.635	[M + H]^+1^	Diosmetin	C_16_ H_12_ O_6_
57	29.407	[M-H]^−1^	Astragaloside IV	C_41_ H_68_ O_14_
58	30.032	[M + H]^+1^	Formononetin	C_16_ H_12_ O_4_
59	30.202	[M + H]^+1^	Medicarpin	C_16_ H_14_ O_4_
60	31.134	[M + H]^+1^	Wilforlide A	C_30_ H_46_ O_3_
61	31.525	[M + H]^+1^	Roburic acid	C_30_ H_48_ O_2_
62	31.661	[M + H]^+1^	5-Hydroxy-6,7-dimethoxylflavone	C_17_ H_14_ O_5_
63	32.78	[M + H]^+1^	Chrysosplenetin B	C_19_ H_18_ O_8_
64	32.913	[M-H]^−1^	Astragaloside II	C_43_ H_70_ O_15_
65	33.378	[M-H]^−1^	Cucurbitacin B	C_32_ H_46_ O_8_
66	35.27	[M+2H]^+2^	Isoastragaloside I	C_45_ H_72_ O_16_
67	35.283	[M-H]^−1^	Dioscin	C_45_ H_72_ O_16_

### Network Pharmacology Analysis Predicted Potential Signaling Pathways of Melanoma Treated by AM and RT

**FIGURE 2:**
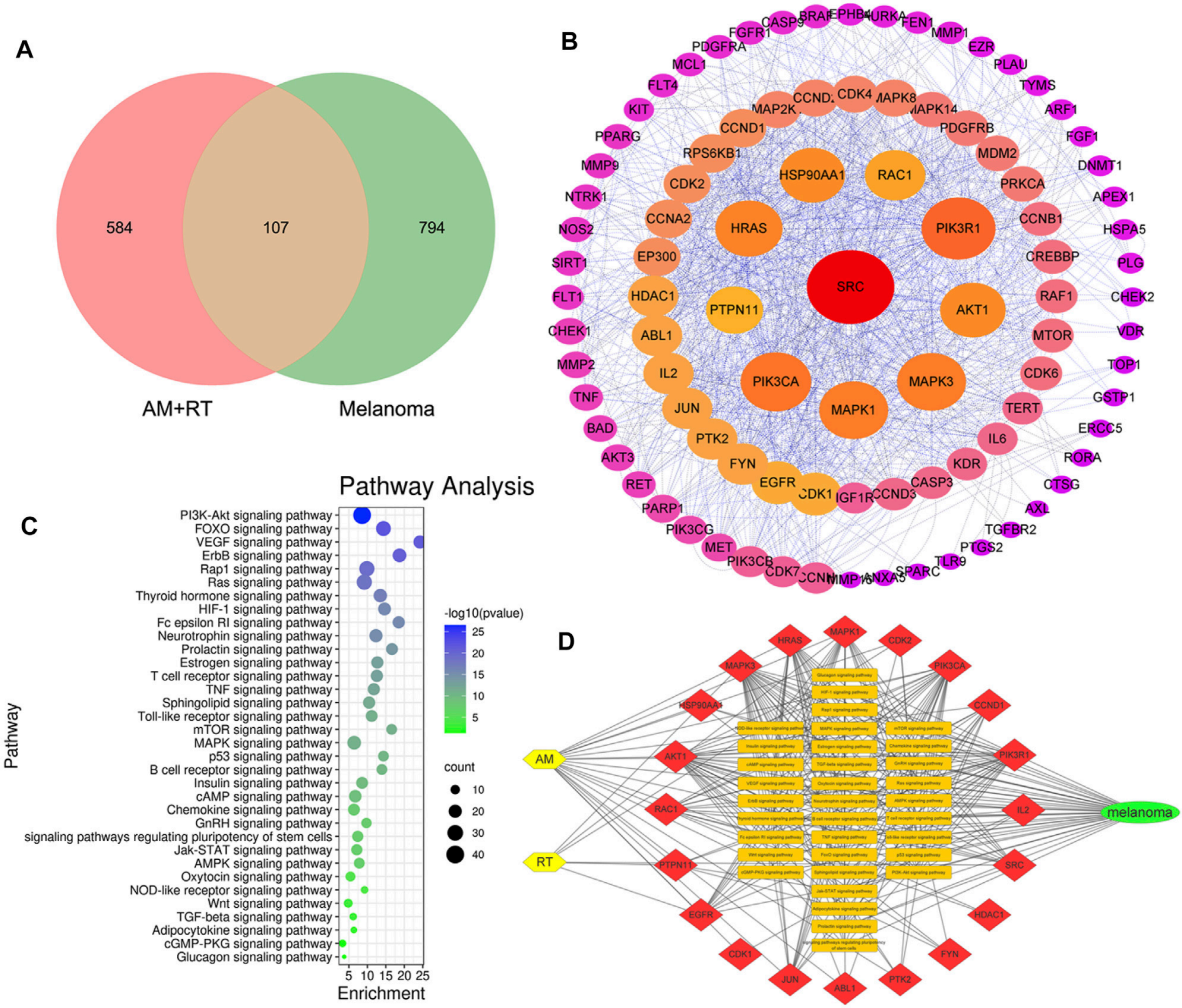
Outcomes of network pharmacology analysis predicted. **(A)** Venn diagram of AM, RT, and MM. **(B)** PPI network of common targets. The layout of the three circles was in a clockwise direction according to the nodes from large to small and red to purple. **(C)** KEGG signaling pathways. The larger the area, the more the counts, and the bluer the color, smaller the *p*-value. **(D)** Drug–target–pathway–disease network. The yellow nodes represent herbs, red of the top 20 targets, orange of 34 pathways, and green of MM.

Overall, 20 active chemical compositions of AM and 2 of RT were obtained via the TCMSP database (Supplementary Table S4). According to aforementioned databases, there are a total of 691 component targets of herbs, and 901 known potential melanoma-related targets were obtained. After summarizing these targets, 107 common targets were obtained, as shown in the Venn diagram in Figure 2A. According to the degree of alignment, the key proteins included SRC, PIK3CA, PIK3R1, Akt1, MAPK1, HRAS, RAC1, MAPK3, HSP90AA1, and PTPN11 (Figure 2B). According to KEGG analysis, 34 signaling pathways were obtained (Figure 2C). The action pathways mainly include PI3K-AKT, FOXO, and VEGF signaling pathways. The drug–target–pathway–disease network was established to reveal the relationship between them (Figure 2D).

### AMD, RTD, and Their Compatibility Inhibit the A375 Cell Proliferation

**FIGURE 3:**
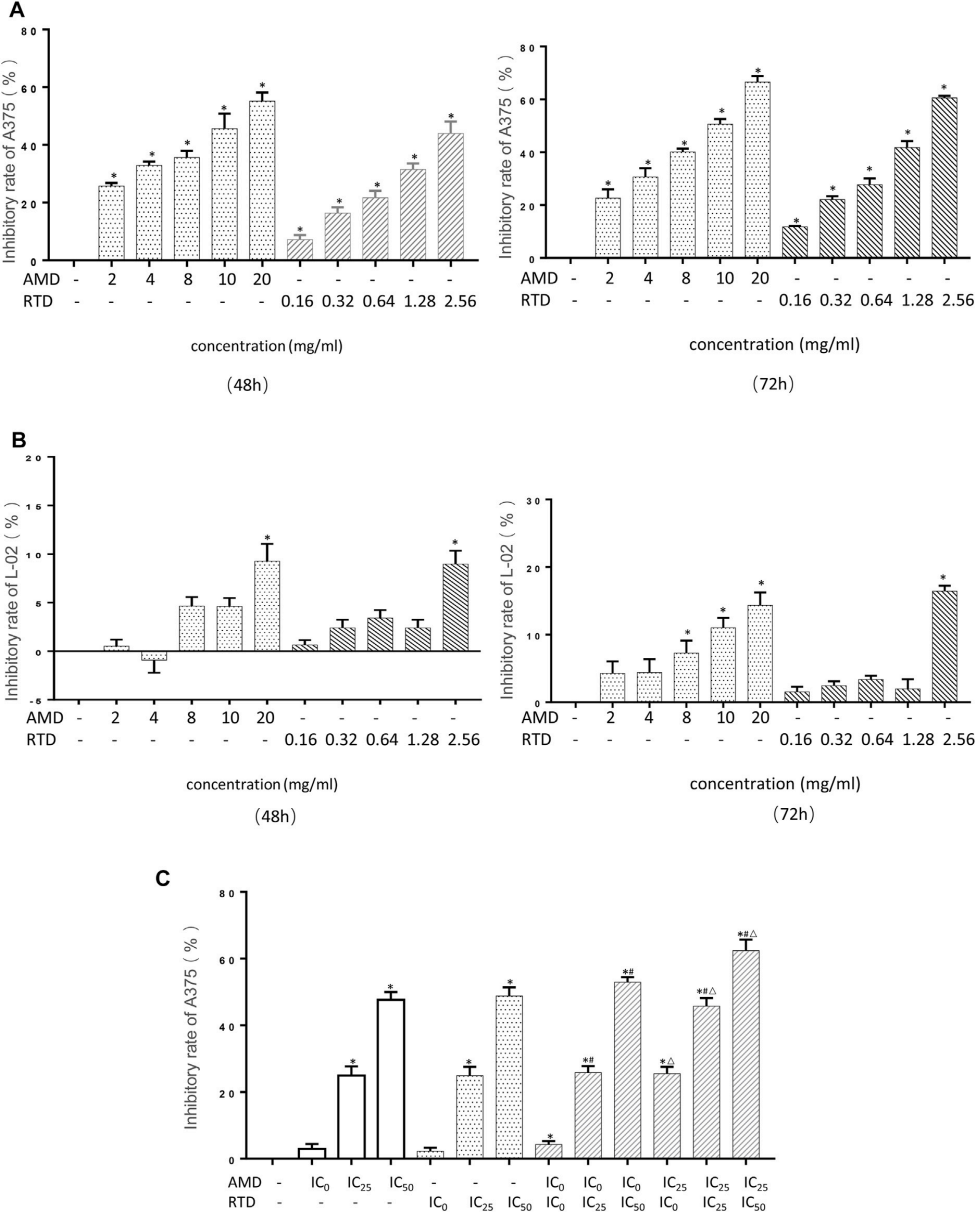
AMD, RTD, and their compatibility inhibited cell proliferation. **(A–B)** Effect on the A375 cells **(A)** and L-02 **(B)** cell proliferation in different concentrations of AMD and RTD for 48 and 72 h. **(C)** Effect of AMD, RTD, and their compatibility on the A375 cell proliferation for 48 h. *n* = 5. ^*∗*^ *p* < 0.05, compared with the control group. ^*#*^ *p* < 0.05, compared with the corresponding AMD group. ^*△*^ *p* < 0.05, compared with the corresponding RTD group.

A375 cell proliferation was significantly inhibited by AMD and RTD in a dose-dependent manner (Figure 3A), while we found there was a low inhibitory effect on L-02 cells (Figure 3B). The corresponding IC_0_, IC_25_, and IC_50_ of each period were calculated (Table 2). They were used to further assess the different effects on A375 cell proliferation with AMD, RTD, and their “cocktail.” The proliferation inhibition rate of “cocktail” groups was higher than those of the corresponding AMD and RTD groups (*p* < 0.05) (Figure 3C). Among all the “cocktail” groups, AMD IC_25_ + RTD IC_50_ (compatibility in a ratio of 5:3) had a stronger inhibition than others on the A375 cell proliferation (Figure 3C).

**TABLE 2:** IC_0_, IC_25_, and IC_50_ of AMD and RTD in each action time.

Drug	Action time (h)	IC_0_ (mg/ml)	IC_25_ (mg/ml)	IC_50_ (mg/ml)
AMD	48	1.1	3.6	14
	72	1.2	4.5	12
RTD	48	0.137	0.863	2.113
	72	0.081	0.769	2.020

### Compatibility of AMD and RTD Could Inhibit Melanoma *In Vivo*

**FIGURE 4:**
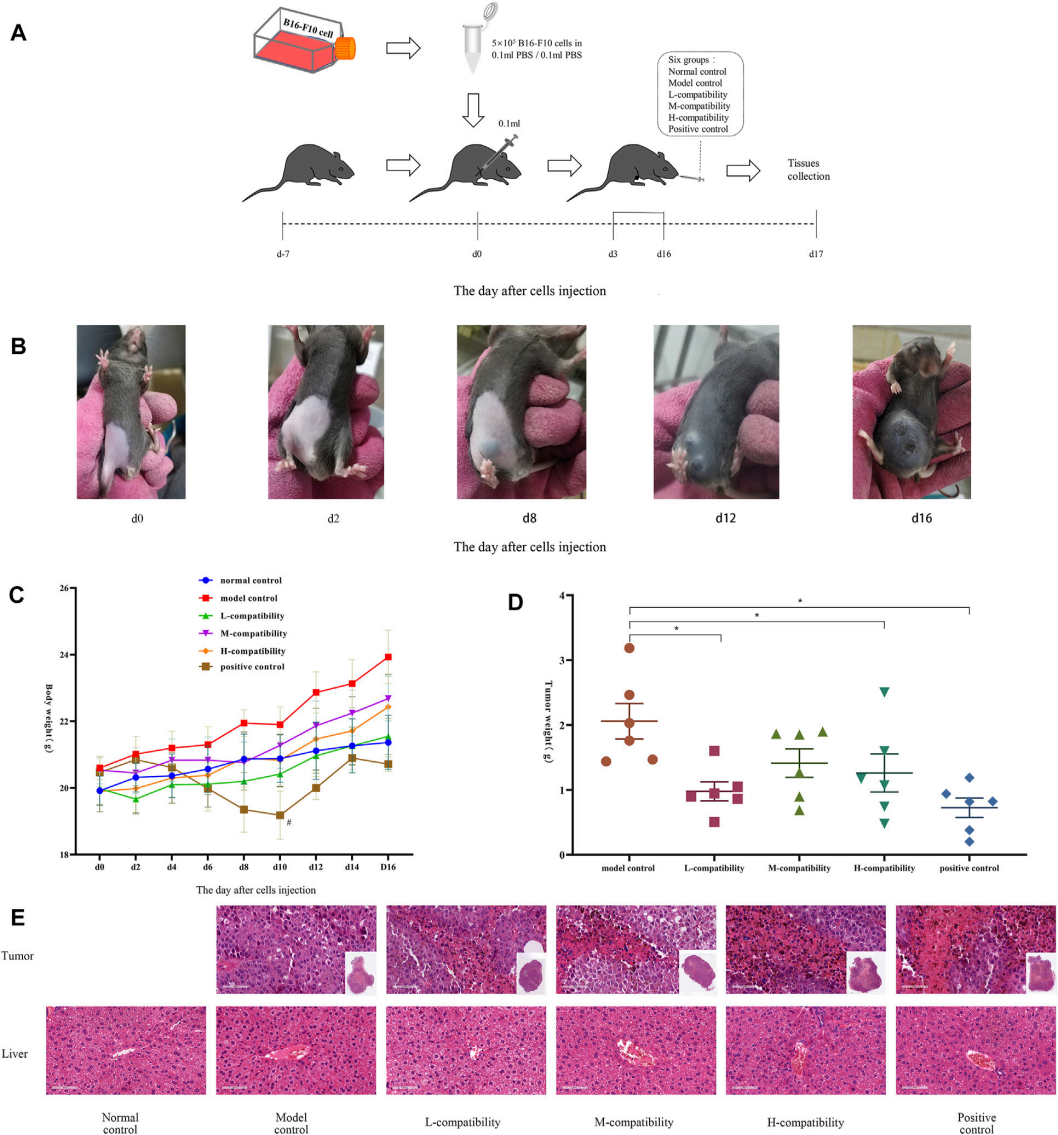
Compatibility of AMD and RTD inhibited B16-F10 melanoma growth. **(A)** Progress of animal experiments. **(B)** B16-F10 tumor in MCG at different time points. **(C)** Body weight of mice at different time points. **(D)** Mean weights of the dissected tumors. **(E)** H&E staining in tumor and liver tissues (200x). *n* = 6. *p < 0.05 versus MCG. ^*#*^ *p* < 0.05, compared with NCG.

We had already confirmed that the compatibility group has better effects than the single drug, and the compatibility has an additive effect. Therefore, in the animal experiment, we directly selected the best compatibility ratio (5:3) to detect its effect on mice with malignant melanoma. After B16-F10 cells were injected 2 days later, the tumor began to form—2 mm in diameter (Figure 4B). Then the mice body weight in most groups showed an increasing trend, while the PCG obviously decreased compared with NCG (Figure 4C), and the spirit and activity of the mice had no obvious change. Compared with MCG, the tumor weight of medication administration groups was decreased significantly (*p* < 0.05) (Figure 4D). The H&E staining results indicated a large area of tumor tissues had disappeared in administration groups (Figure 4E), compared with the MCG. Interestingly, there was no significant difference in the pathological sections of liver tissues in all groups.

### AMD, RTD, and Their Compatibility Affect the A375 Cell Cycle and Apoptosis

**FIGURE 5:**
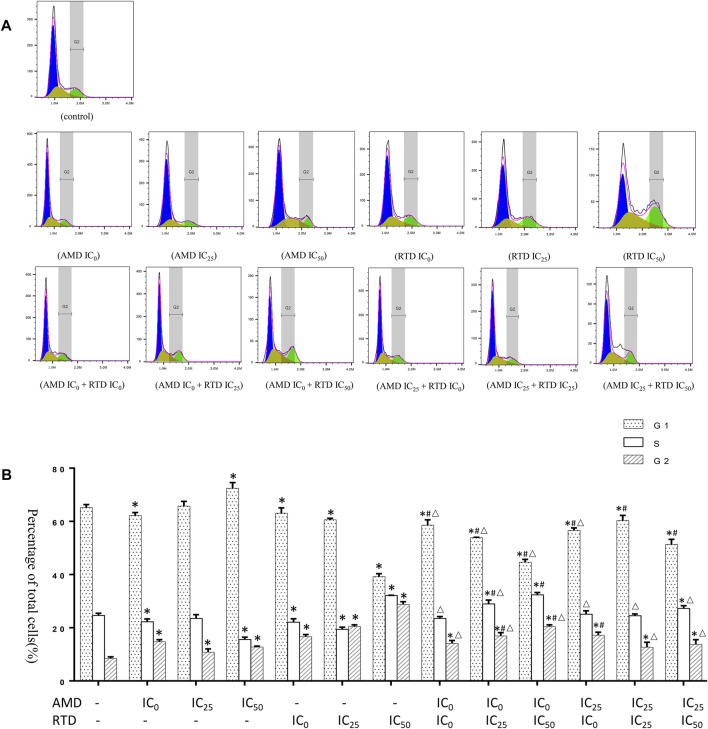
Effects of AMD, RTD, and their compatibility on A375 cell cycle. **(A)** Flow analysis result of A375 cell cycle. **(B)** Statistical histogram of A375 cell cycle resulst. *n* = 3. ^*∗*^ *p* < 0.05, compared with the control group. ^*#*^ *p* < 0.05, compared with the corresponding AMD group. ^*△*^ *p* < 0.05, compared with the corresponding RTD group.

**FIGURE 6:**
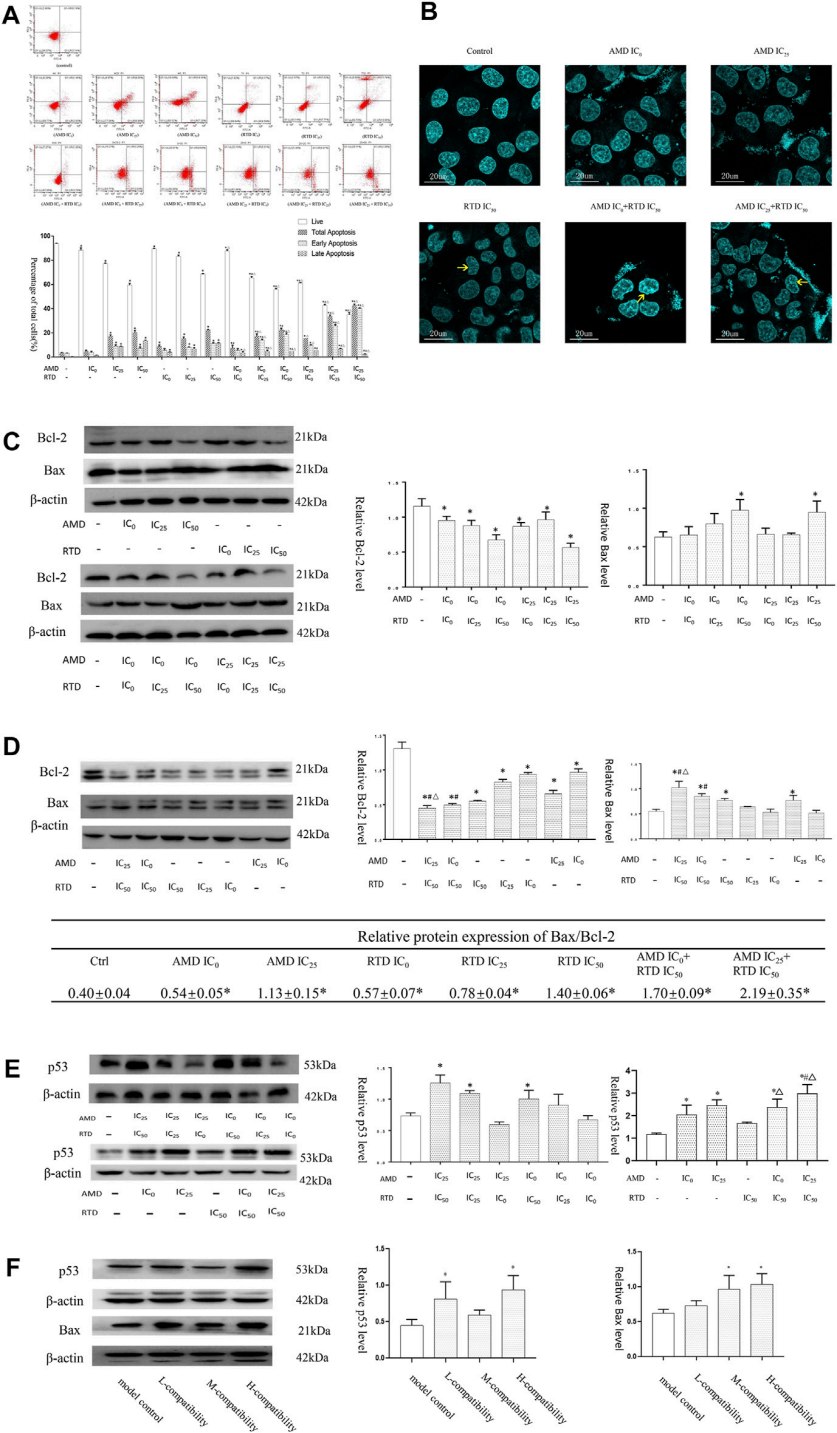
AMD, RTD, and their compatibility promoted MM apoptosis. **(A–B)** Effects of AMD, RTD, and their compatibility on A375 cell apoptosis. **(C–D)** Western blot analysis and relative quantification levels of Bcl-2 and Bax expression with herb treatment for 48 h (C) and 72 h **(D)** of A375 cells. **(E)** Western blot analysis and relative quantification levels of p53 expression of A375 cells with herb treatment for 72 h. *n* = 3. ^*∗*^ *p* < 0.05, compared with the control group. ^*#*^ *p* < 0.05, compared with the corresponding AMD group. ^*△*^ *p* < 0.05, compared with the corresponding RTD group. **(F)** Western blot analysis and relative quantification levels of p53 and Bax expression of the mice tumors. ^*∗*^ *p* < 0.05, compared with MCG.

To investigate the mechanism of tumor growth and cell proliferation inhibition, we further analyzed the cell cycle and apoptosis *in vitro*. AMD, RTD, and their “cocktail” could play an inhibitory role in cell proliferation through the induction of cell cycle arrest (Figure 5). Furthermore, AMD, RTD, and their “cocktail” could promote A375 cell apoptosis in a dose-dependent manner, and the numbers of early and total apoptotic cells increased significantly in the “cocktail” groups, which showed an additive effect of compatibility (Figure 6A). Hoechst staining results showed that the most nuclear membranes in groups RTD IC_50_ (AMD IC_0_+RTD IC_50_) and (AMD IC_25_ + RTD IC_50_) were obviously wrinkled, and the chromatin in nuclei was condensed (Figure 6B). Meanwhile, the herbs could reduce Bcl-2 expression and enhance the Bax protein level, with a ratio of Bax/Bcl-2 significantly increased in a dose-dependent manner (Figures 6C,D), and the “cocktail” groups had better effects, especially (AMD IC_0_+RTD IC_50_) and (AMD IC_25_ + RTD IC_50_). In addition, we found that the herbs could also increase p53 protein expression, and group (AMD IC_25_ + RTD IC_50_) had the best effect (Figure 6E). *In vivo*, the compatibility treated in the melanoma model could also promote p53 and Bax protein expression (Figure 6F).

### Cell Migration Could Be Inhibited in A375 Cells Treated With AMD, RTD, and Their Compatibility

**FIGURE 7:**
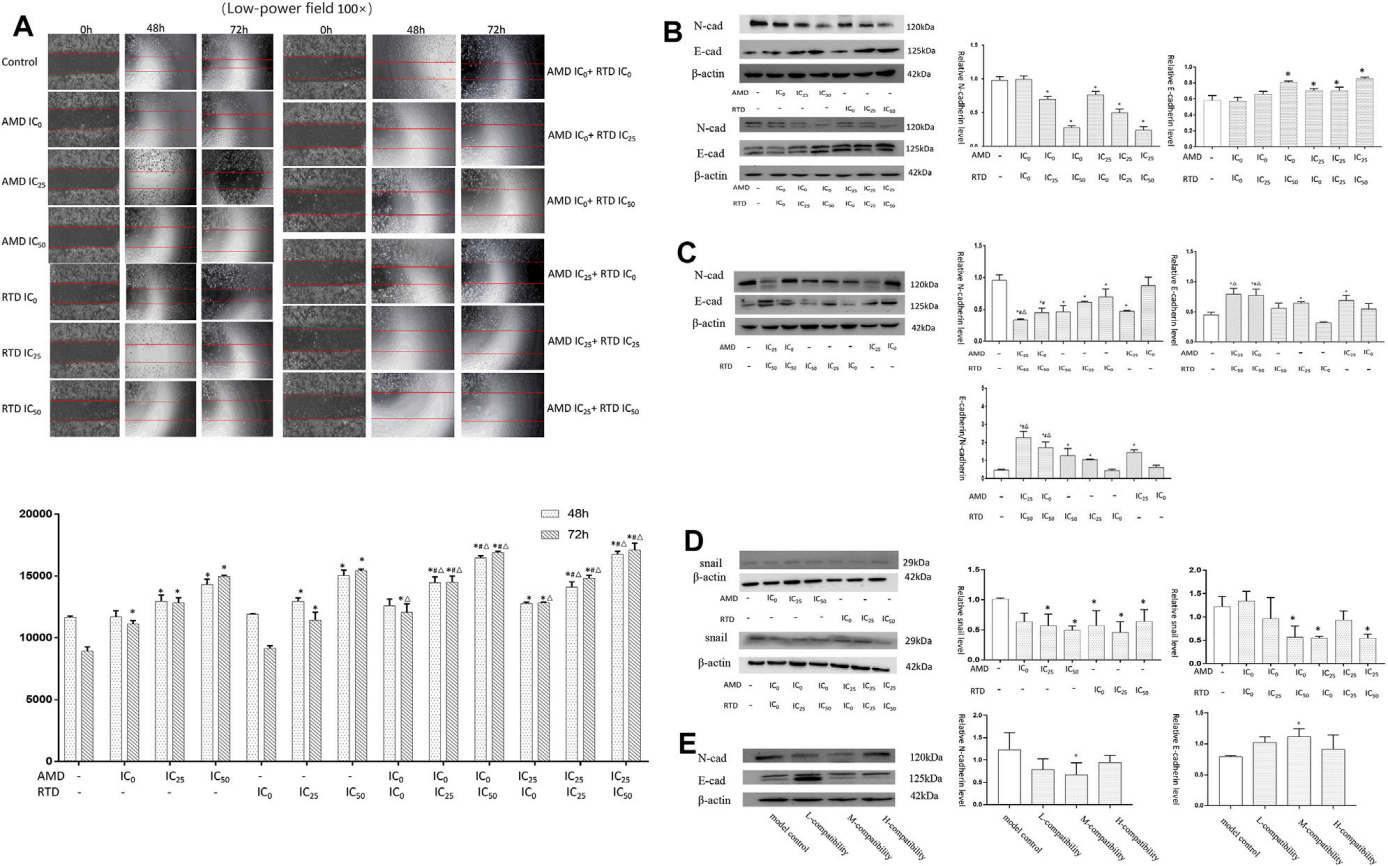
AMD, RTD, and their compatibility inhibited MM migration. **(A)** Effects on A375 cell migration with AMD, RTD, and their compatibility for 48 and 72 h. **(B–C)** Western blotting analysis and relative quantification levels of N-cadherin and E-cadherin expression of A375 cells with herb treatment for 48 h **(B)** and 72 h **(C)**. **(D)** Western blotting analysis and relative quantification levels of snail expression of A375 cells with herb treatment for 48 h. *n* = 3. ^*∗*^ *p* < 0.05, compared with the control group. ^*#*^ *p* < 0.05, compared with the corresponding AMD group. ^*△*^ *p* < 0.05, compared with the corresponding RTD group. **(E)** Western blot analysis and relative quantification levels of N-cadherin and E-cadherin expression of the mice tumors. ^*∗*^ *p* < 0.05, compared with MCG.

The wound healing assay showed that the control group suffered a closing tendency of a scratch wound and the cell number increased, while the others led to slower closing of a scratch wound with statistical difference (Figure 7A). Thus, AMD, RTD, and their “cocktail” could inhibit the A375 cell migration in a dose-dependent manner. The “cocktail” groups had a stronger cell migration inhibition, especially groups (AMD IC_0_+RTD IC_50_) and (AMD IC_25_ + RTD IC_50_).

Furthermore, in treatment groups of A375 cells, especially “cocktail” groups (AMD IC_0_ + RTD IC_50_) and (AMD IC_25_ + RTD IC_50_), E-cadherin increased and N-cadherin decreased, resulting in an increased ratio of E-cadherin/N-cadherin (Figures 7B,C). In addition, snail expression of A375 cells decreased (Figure 7D). *In vivo*, E-cadherin increased and N-cadherin decreased in tumor tissues treated by the compatibility (Figure 7E).

### AMD, RTD, and Their Compatibility Affect the LDH, SOD, and MDA Content *in vitro* and *vivo*

**FIGURE 8:**
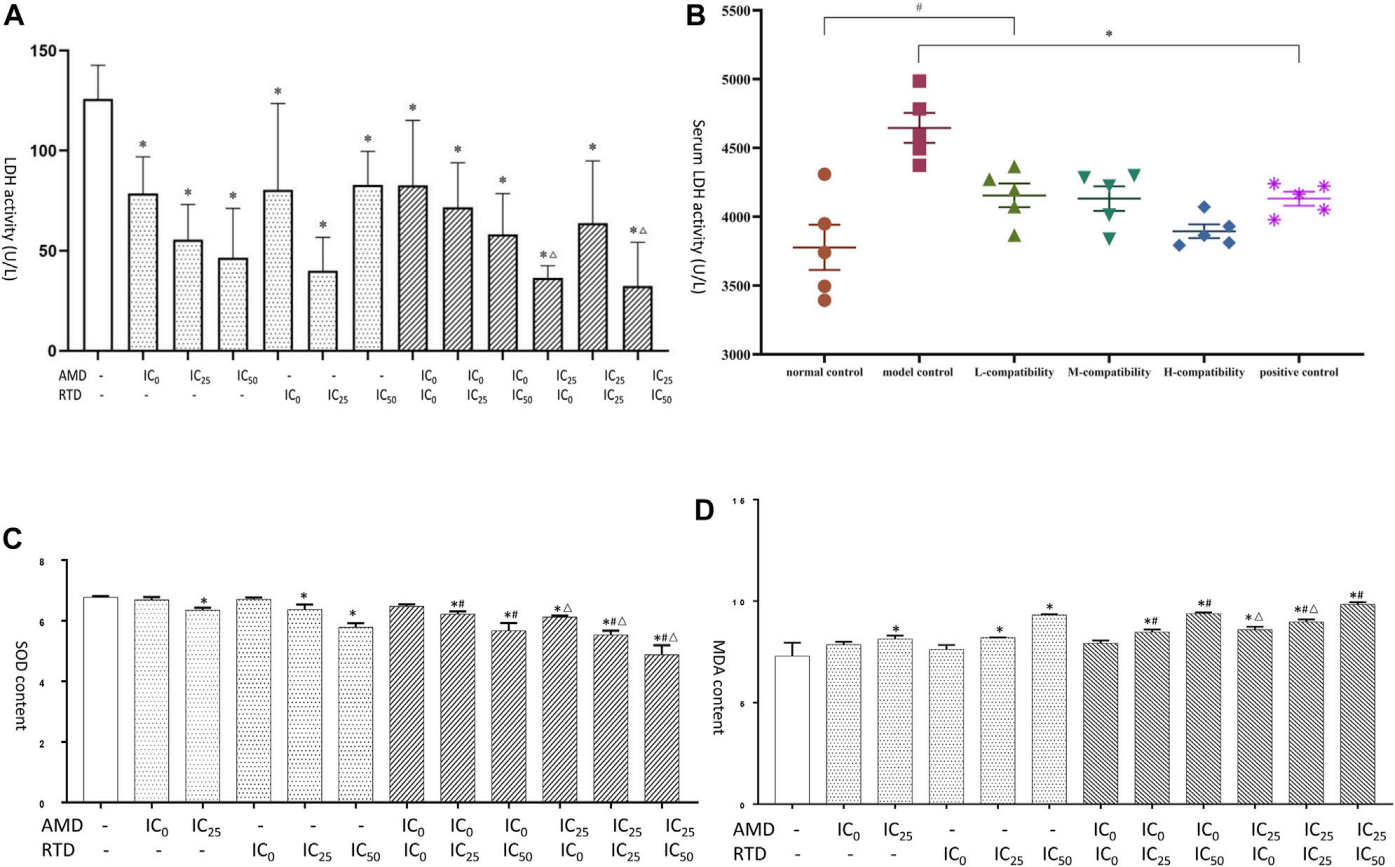
AMD, RTD, and their compatibility affect LDH, SOD, and MDA. **(A)** Effects of AMD, RTD, and their compatibility on LDH activity of A375 cells. **(C–D)** Effects of AMD, RTD, and their compatibility on SOD **(C)** and MDA **(D)** content of A375 cells. *n* = 3. ^*∗*^ *p* < 0.05, compared with the control group. ^*#*^ *p* < 0.05, compared with the corresponding AMD group. ^*△*^ *p* < 0.05, compared with the corresponding RTD group. **(B)** Effects of serum of different mice on LDH activity. *n* = 5. ^*∗*^ *p* < 0.05, compared with MCG. ^*#*^ *p* < 0.05, compared with NCG.

LDH is a key enzyme involved in tumor glycolysis and associated with tumor proliferation and metastasis. SOD and MDA are markers of oxidation and antioxidation and are also related to tumor apoptosis and metastasis. Therefore, we examined their expression. After treatment with AMD, RTD, and their “cocktail” for 48 h in A375 cells, the LDH activity of herb-only groups decreased, and the “cocktail” groups (AMD IC_25_ + RTD IC_0_) and (AMD IC_25_ + RTD IC_50_) had an additive effect with a statistical difference (Figure 8A). *In vivo*, compared with the NCG, the LDH activity of the MCG significantly increased, and that in other groups decreased (Figure 8B). In addition, the MDA content increased and the SOD activity decreased, in a dose-dependent manner. The “cocktail” groups, especially (AMD IC_0_+RTD IC_50_) and (AMD IC_25_ + RTD IC_50_), had an additive effect (Figure 8C, D).

### AMD, RTD, and Their Compatibility Could Alter the Expression of Protein (Akt and p-Akt) in A375 Cells

**FIGURE 9:**
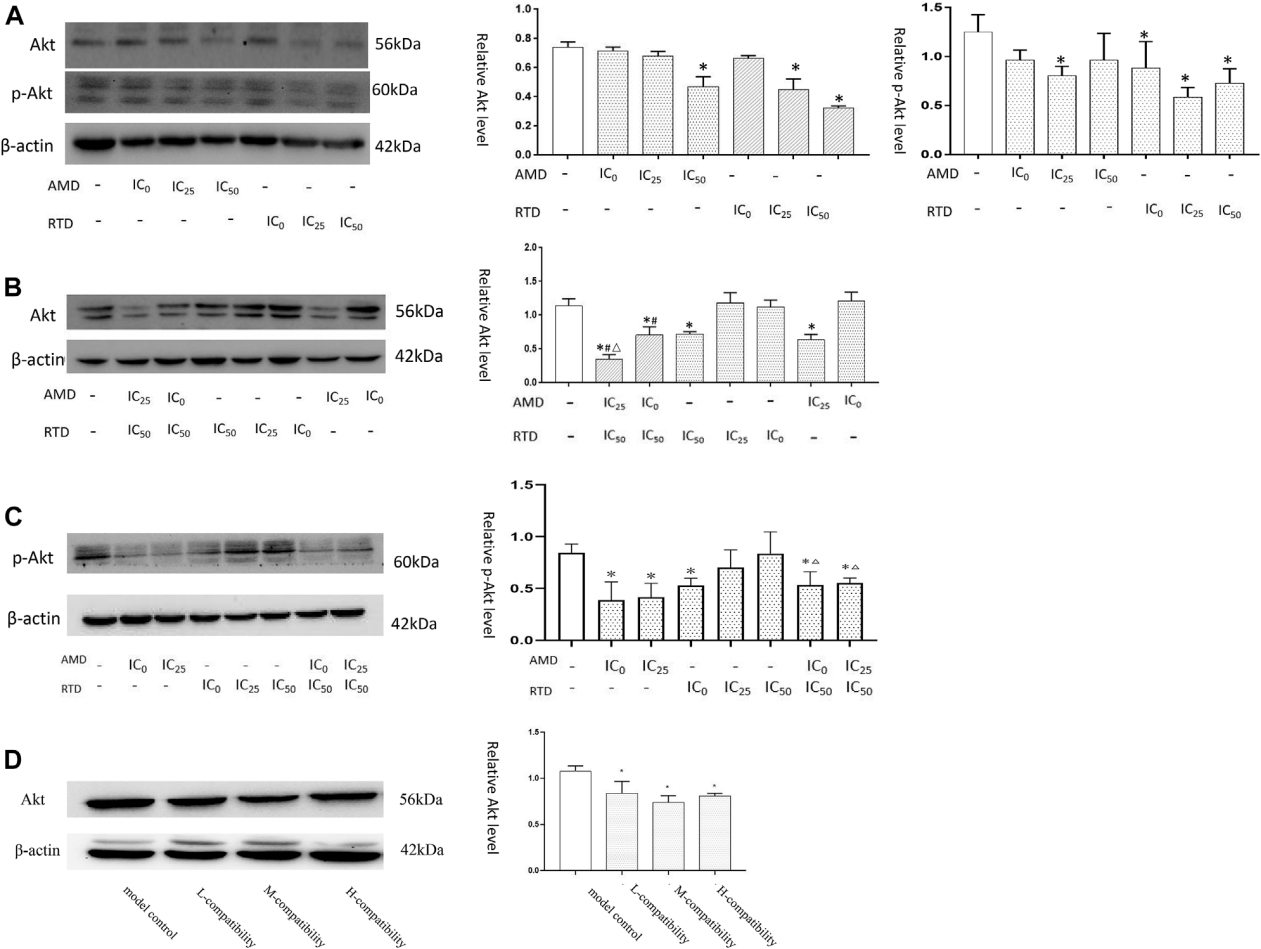
AMD, RTD, and their compatibility affect Akt-related protein expression. **(A)** Western blotting analysis and relative quantification levels of Akt and p-Akt expression of A375 cells with herb treatment for 48 h **(A)** and 72 h **(B)**. **(C)** Western blotting analysis and relative quantification levels of p-Akt expression of A375 cells with herb treatment for 72 h. *n* = 3. ^*∗*^ *p* < 0.05, compared with the control group. ^*#*^ *p* < 0.05, compared with the corresponding AMD group. ^*△*^ *p* < 0.05, compared with the corresponding RTD group. **(D)** Western blot analysis and relative quantification levels of Akt expression of the tissues. ^*∗*^ *p* < 0.05, compared with MCG.

*In vitro*, we found that after herb treatment for 48 h, certain doses of AMD and RTD led to a decrease in Akt and p-Akt expression. For 72 h, herbs, especially “cocktail” groups (AMD IC_0_+RTD IC_50_) and (AMD IC_25_ + RTD IC_50_), could reduce the expression of Akt and p-Akt. *In vivo*, their compatibility also could reduce the Akt protein expression in melanoma tissues (Figure 9D).

## Discussion

As important qi-tonifying herbs are to be widely used in clinics, AM and RT were originally published in the Chinese medical ancient book Shen Nong’s Herbal Classic. Currently, there is much evidence that suggested that AM and RT have antitumor, anti-inflammatory, and antiviral effects, with low expenditure and few side effects (Zhou et al., 2007; Li et al., 2020). LC-MS and network pharmacology analysis pointed out these herbs have multiple components and multiple targets which may exert an antitumor effect. Compared with AM and RT, the cocktail of them possesses some different compounds, such as berberine, which may promote the effects on melanoma and against tumor (Huang et al., 2021). According to our early studies, AMD and RTD possess an antitumor effect because they can inhibit MM growth and metastasis by inhibiting the proliferation and migration of mouse malignant melanoma B16 cells (Zhang et al., 2020). Furthermore, the PI3K-Akt signaling pathway may play a crucial role in the treatment, and the aforementioned data, which we researched, also confirmed that AMD and RTD could have a restraining effect on mice melanoma and human malignant melanoma A375 cells.

Tumor occurrence is closely associated with unrestricted proliferation, which is also one of the root causes of tumor development. Based on that, directly killing tumor cells or inhibiting proliferation is the main way for antitumor drugs to take effect. In this study, it was confirmed that AMD and RTD could both play a role in inhibiting the A375 cell proliferation while not affecting or slightly affecting L-02 cells. This made it clear that AMD and RTD are noncytotoxic agents at a certain dose, which has great significance in the promotion of their application. Furthermore, *in vivo*, the best compatibility of AMD and RTD could inhibit melanoma growth while not affecting their livers.

Combining with the results of the MTT assay, we found that AMD (below 4 mg/ml) and RTD (below 1.28 mg/ml) had no effect on the normal cell proliferation but caused cell cycle arrest in different phases of A375 cells. Cells that arrest in G_1_ and G_2_ phases may return to a stationary state (G_0_ phase) or undergo apoptosis when the DNA damage is too serious to be repaired, with an irregular nuclear membrane, condensed chromatin, and crumpled nuclear membrane (Hustedt and Durocher 2016; Lezaja and Altmeyer 2018). These changes could be the featured phenomena of early apoptosis. This research showed that AMD, RTD, and their compatibility could promote early apoptosis in A375 cells, especially the “cocktail” groups.

Antitumor drugs could induce apoptosis by promoting the expression of Bax and p53, or inhibiting the expression of Bcl-2. Similarly, we demonstrated that AMD, RTD, and their “cocktail” could promote apoptosis in A375 cells via modulating Bax, Bcl-2, and p53 expression. *In vivo*, the best compatibility of AMD and RTD also could influence tumors via promoting p53 expression or inhibiting Bcl-2 expression. In summary, we could know that AMD and RTD may achieve a better therapeutic effect on MM by inhibiting proliferation, regulating the cell cycle, and inducing apoptosis. The “cocktail” had an additive antitumor effect.

MM had high metastatic potential. The epithelial–mesenchymal transition (EMT) was a crucial step in the cell invasion and metastasis, with two makers of E-cadherin and N-cadherin. It was confirmed that the upregulation of E-cadherin and suppression of N-cadherin were effective in inhibiting cell migration and invasion (Fenouille et al., 2012; John et al., 2012). In our study, AMD and RTD could inhibit the A375 cell migration by affecting the expression of E-cadherin, N-cadherin, and snail protein. Their “cocktail” also could regulate the expression level of E-cadherin and N-cadherin in mice tissues. Additionally, one study found that *Astragalus Membranaceus*–*Curcuma zedoaria* can obviously inhibit tumor cell migration; this was similar to the results from our study (Tan et al., 2019). “Cocktail” of AMD and RTD could markedly inhibit the A375 cell migration, which showed an additive effect.

The growth of cancer cells required glycolysis, and excessive glycolysis could promote cell proliferation and metastasis (Vander Heiden et al., 2009). LDH was a key enzyme during glycolysis to promote malignant behavior via the activation of EMT in cancer cells (Jurisic et al., 2015; Hou et al., 2019), and drugs could decrease glycolysis by reducing the LDH activity, thereby inhibiting cell proliferation and migration. We found that AMD and RTD could reduce the LDH activity of A375 cells, which might be one of the mechanisms for inhibiting the A375 proliferation and migration. The serum LDH of the MCG was higher than that of the NCG, and the abnormal increase in LDH was reduced in each administration group. It meant that the “cocktail” of AMD and RTD could effectively inhibit melanoma metastasis.

SOD was an important antioxidant biomarker in the organism, and MDA was frequently used as an indicator of cell membrane oxidative damage. Studies had demonstrated that antioxidants can be effective against cell apoptosis and promote the migration of MM cells (Le Gal et al., 2015). In our study, we found that the activity of SOD was decreased, whereas the MDA content was increased in drug groups of A375 cells, especially the “cocktail” groups. This resulted in increased oxidative stress and cell damage, leading to apoptosis.

Akt was an important oncogene closely related to cell proliferation, migration, and metabolism, and there was a significant difference in Akt expressions between MM and common pigmented nevi. The death of MM cells could be promoted by reducing Akt activity and inhibiting Akt phosphorylation (Bhattacharya et al., 2011; Dang et al., 2018). Activated Akt played an important role in both cell apoptosis and migration. It could not only regulate apoptosis by affecting Bax and Bcl-2 expression but also inhibit the expression of E-cadherin to promote EMT occurrence (Qu et al., 2015; Qin et al., 2017). These all had been confirmed to occur in MM progression. In this study, the results showed that AMD and RTD, especially their “cocktail,” could reduce the expressions of Akt and p-Akt protein to restrain their activity and phosphorylation. It may be the mechanism for AMD, RTD, and their “cocktail” to regulate the proliferation, cell cycle, apoptosis, and migration of A375 cells. Moreover, activated Akt can also affect the production of ROS, thereby affecting the survival of MM cells (Hambright et al., 2015).

**FIGURE 10:**
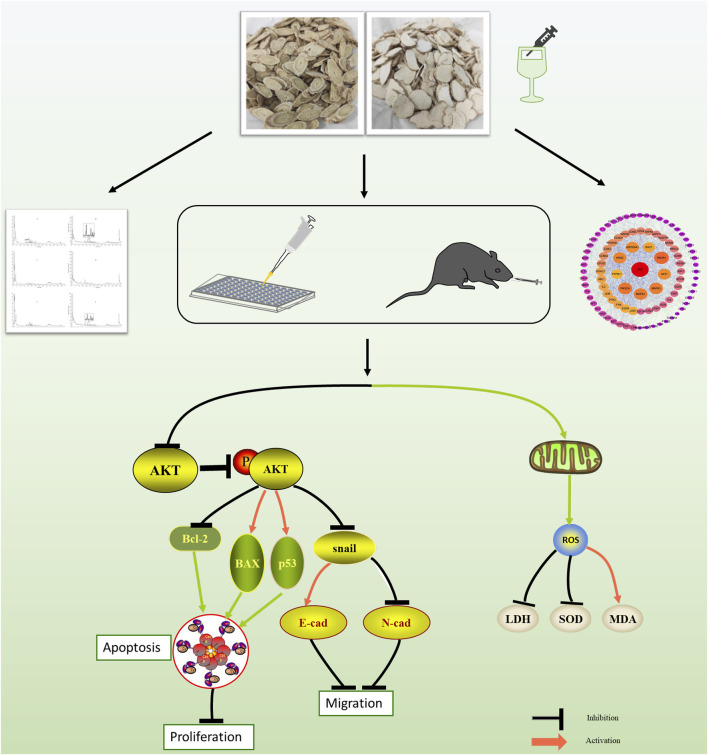
Progress of this study and the effects of AMD, RTD, and their “cocktail” on the Akt-related signaling pathway *in vitro* and *in vivo*.

In conclusion, the present study explored the antitumor effect on MM *in vitro* and *in vivo* of AMD, RTD, and their compatibility. The results clarified that AMD, RTD, and their “cocktail” could exert an antitumor effect by regulating the Akt-related signaling pathway to influence the cell proliferation cycle, apoptosis, migration, LDH activity, SOD activity, and MDA content (Figure 10). The additive effect of compatibility *in vitro* was confirmed, and the best compatibility group obtained by *in vitro* experiments could indeed inhibit the growth of mouse melanoma *in vivo*. These provide the experimental basis and new ideas for the clinical treatment with TCM in MM.

## Data Availability

The raw data supporting the conclusions of this article are available from corresponding author upon reasonable request.
