# Supplementary material for: Cocktail of Astragalus Membranaceus and Radix Trichosanthis Suppresses Melanoma Tumor Growth and Cell Migration Through Regulation of Akt-Related Signaling Pathway
Source: Front Pharmacol. 2022 Jun 1;13:880215. doi: 10.3389/fphar.2022.880215 (PMC9198299; doi:10.3389/fphar.2022.880215)
Supplement: Supplementary file 1 [file DataSheet1.docx]

**Supplementary documents：**

Figure S1

Research process of mechanism of AM and RT in melanoma based on network pharmacology.

**
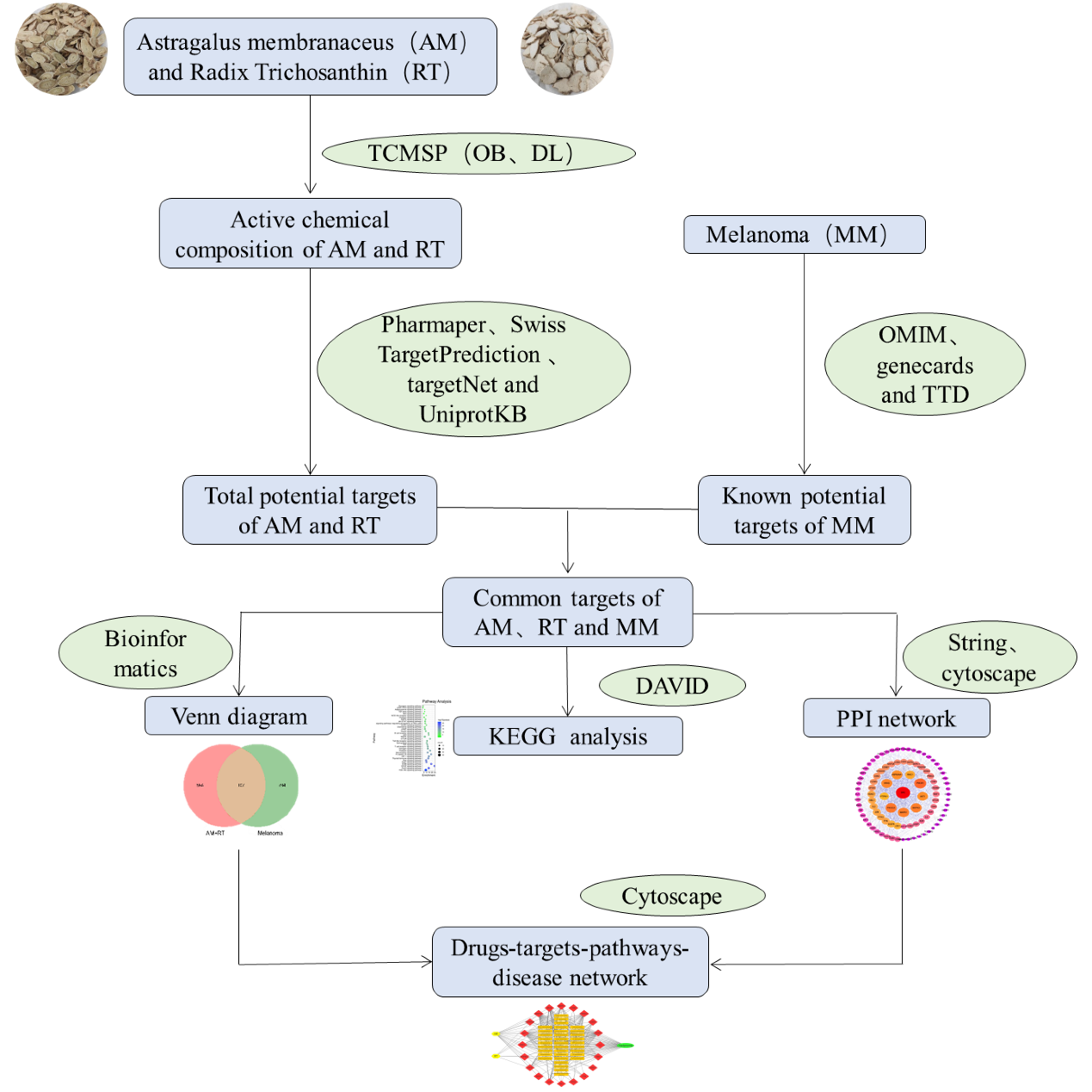
**

Table S1

Gradient elution conditions.

| **Time（min）** | **A：acetonitrile(%)** | **B：water with 0.1% formic acid(%)** | **flow rate（mL/min）** |
| --- | --- | --- | --- |
| 0 | 0 | 100 | 0.2 |
| 10 | 0 | 100 | 0.2 |
| 20 | 30 | 70 | 0.2 |
| 25 | 40 | 60 | 0.2 |
| 30 | 50 | 50 | 0.2 |
| 40 | 70 | 30 | 0.2 |
| 45 | 100 | 0 | 0.2 |
| 60 | 100 | 0 | 0.2 |
| 60.1 | 0 | 100 | 0.2 |
| 70 | 0 | 100 | 0.2 |

Table S2

Information of compounds of AMD detected by LC-ESI-MS/MS in different Ion mode.

| **No.** | **RT [min]** | **Ion mode** | **Name** | **Formula** |
| --- | --- | --- | --- | --- |
| 1 | 1.59 | [M+H]^+1^ | L-Threonine | C_4_ H_9_ N O_3_ |
| 2 | 1.595 | [M-H]^-1^ | L-Glutamic acid | C_5_ H_9_ N O_4_ |
| 3 | 1.717 | [M+H]^+1^ | Trigonelline HCl | C_7_ H_7_ N O_2_ |
| 4 | 1.736 | [M+H]^+1^ | 2-Pyrrolidinecarboxylic acid | C_5_ H_9_ N O_2_ |
| 5 | 1.769 | [M+H]^+1^ | Adenine | C_5_ H_5_ N_5_ |
| 6 | 2.453 | [M+H]^+1^ | L-Valine | C_5_ H_11_ N O_2_ |
| 7 | 2.751 | [M+H]^+1^ | Nicotinic acid | C_6_ H_5_ N O_2_ |
| 8 | 3 | [M+H]^+1^ | Cytosine | C_4_ H_5_ N_3_ O |
| 9 | 3.403 | [M+H]^+1^ | Nicotinamide | C_6_ H_6_ N_2_ O |
| 10 | 3.584 | [M-H]^-1^ | Citric acid | C_6_ H_8_ O_7_ |
| 11 | 4.065 | [M-H]^-1^ | Maleic acid | C_4_ H_4_ O_4_ |
| 12 | 5.766 | [M-H]^-1^ | Uridine | C_9_ H_12_ N_2_ O_6_ |
| 13 | 17.798 | [M-H]^-1^ | Asperulosidic acid | C_18_ H_24_ O_12_ |
| 14 | 18.346 | [M-H]^-1^ | L-Tryptophan | C_11_ H_12_ N_2_ O_2_ |
| 15 | 18.708 | [M+H]^+1^ | Complanatuside | C_28_ H_32_ O_16_ |
| 16 | 19.038 | [M-H]^-1^ | 4-Hydroxybenzoic acid | C_7_ H_6_ O_3_ |
| 17 | 19.157 | [M-H]^-1^ | Gentiopicrin | C_16_ H_20_ O_9_ |
| 18 | 19.505 | [M-H]^-1^ | 4-Methoxyphenylacetic acid | C_9_ H_10_ O_3_ |
| 19 | 19.819 | [M+H]^+1^ | Oroxin B | C_27_ H_30_ O_15_ |
| 20 | 20.064 | [M-H]^-1^ | Protocatechuic acid | C_7_ H_6_ O_4_ |
| 21 | 20.177 | [M+H]^+1^ | Orsellinic acid ethyl ester | C_10_ H_12_ O_4_ |
| 22 | 20.267 | [M-H]^-1^ | Sibiricose A5 | C_22_ H_30_ O_14_ |
| 23 | 20.51 | [M+H]^+1^ | Sarracenin | C_11_ H_14_ O_5_ |
| 24 | 20.58 | [M+H]^+1^ | Abscisic acid | C_15_ H_20_ O_4_ |
| 25 | 20.928 | [M-H]^-1^ | Tenuifoliside A | C_31_ H_38_ O_17_ |
| 26 | 20.975 | [M+H]^+1^ | 7-Methoxycoumarin | C_10_ H_8_ O_3_ |
| 27 | 21.644 | [M+H]^+1^ | 2-Hydroxy-4-methoxybenzaldehyde | C_8_ H_8_ O_3_ |
| 28 | 21.672 | [M-H]^-1^ | Sinapic acid | C_11_ H_12_ O_5_ |
| 29 | 21.778 | [M+H]^+1^ | Calycosin-7-O-β-D-glucoside | C_22_ H_22_ O_10_ |
| 30 | 21.805 | [M-H]^-1^ | Bruceine A | C_26_ H_34_ O_11_ |
| 31 | 21.935 | [M-H]^-1^ | Iridin | C_24_ H_26_ O_13_ |
| 32 | 22.029 | [M-H]^-1^ | Isoliquiritin | C_21_ H_22_ O_9_ |
| 33 | 22.068 | [M+H]^+1^ | 3,5-Dimethoxy-4-hydroxybenzaldehyde | C_9_ H_10_ O_4_ |
| 34 | 22.485 | [M+H]^+1^ | Genistin | C_21_ H_20_ O_10_ |
| 35 | 23.185 | [M+H]^+1^ | Tectoridin | C_22_ H_22_ O_11_ |
| 36 | 23.194 | [M-H]^-1^ | Phloridzin | C_21_ H_24_ O_10_ |
| 37 | 23.215 | [M+H]^+1^ | Isosakuranetin | C_16_ H_14_ O_5_ |
| 38 | 23.217 | [M+H]^+1^ | Isosakuranin | C_22_ H_24_ O_10_ |
| 39 | 23.543 | [M-H]^-1^ | Azelaic acid | C_9_ H_16_ O_4_ |
| 40 | 23.564 | [M+H]^+1^ | Jatrorrhizine | C_20_ H_19_ N O_4_ |
| 41 | 23.908 | [M-H]^-1^ | Salicylic acid | C_7_ H_6_ O_3_ |
| 42 | 24.089 | [M+H]^+1^ | 6''-O-Acetylglycitin | C_24_ H_24_ O_11_ |
| 43 | 24.241 | [M+H]^+1^ | Baicalin | C_21_ H_18_ O_11_ |
| 44 | 24.264 | [M+H]^+1^ | Ononin | C_22_ H_22_ O_9_ |
| 45 | 24.557 | [M+H]^+1^ | Ligustilide | C_12_ H_14_ O_2_ |
| 46 | 25.002 | [M+H]^+1^ | Palmatine | C_21_ H_21_ N O_4_ |
| 47 | 25.032 | [M+H]^+1^ | 4',7-Di-O-methylnaringenin | C_17_ H_16_ O_5_ |
| 48 | 25.034 | [M+H]^+1^ | o-Veratraldehyde | C_9_ H_10_ O_3_ |
| 49 | 25.258 | [M+H]^+1^ | Epiberberine | C_20_ H_17_ N O_4_ |
| 50 | 25.297 | [M+H]^+1^ | Scutellarin methyl ester | C_22_ H_20_ O_12_ |
| 51 | 25.403 | [M-H]^-1^ | Isomucronulatol 7-O-glucoside | C_23_ H_28_ O_10_ |
| 52 | 25.406 | [M+H]^+1^ | Benzoic acid | C_7_ H_6_ O_2_ |
| 53 | 25.542 | [M+H]^+1^ | Liquiritigenin | C_15_ H_12_ O_4_ |
| 54 | 25.609 | [M+H]^+1^ | Oroxylin A-7-O-β-D-glucuronide | C_22_ H_20_ O_11_ |
| 55 | 25.961 | [M+H]^+1^ | Calycosin | C_16_ H_12_ O_5_ |
| 56 | 26.078 | [M-H]^-1^ | Rhaponticin | C_21_ H_24_ O_9_ |
| 57 | 26.199 | [M+H]^+1^ | Wogonoside | C_22_ H_20_ O_11_ |
| 58 | 26.347 | [M+H]^+1^ | Pectolinarigenin | C_17_ H_14_ O_6_ |
| 59 | 26.47 | [M+H]^+1^ | Glycitin | C_22_ H_22_ O_10_ |
| 60 | 27.844 | [M-H]^-1^ | Genistein | C_15_ H_10_ O_5_ |
| 61 | 27.999 | [M+H]^+1^ | Artemisinic acid | C_15_ H_22_ O_2_ |
| 62 | 28.228 | [M-H]^-1^ | Hydroxygenkwanin | C_16_ H_12_ O_6_ |
| 63 | 28.416 | [M+H]^+1^ | Dehydrotrametenolic acid | C_30_ H_46_ O_3_ |
| 64 | 28.652 | [M+H]^+1^ | Diosmetin | C_16_ H_12_ O_6_ |
| 65 | 28.798 | [M+H]^+1^ | 2-Adamantanone | C_10_ H_14_ O |
| 66 | 29.41 | [M-H]^-1^ | Astragaloside IV | C_41_ H_68_ O_14_ |
| 67 | 30.052 | [M+H]^+1^ | Formononetin | C_16_ H_12_ O_4_ |
| 68 | 30.219 | [M+H]^+1^ | Medicarpin | C_16_ H_14_ O_4_ |
| 69 | 31.156 | [M-H]^-1^ | Astragaloside II | C_43_ H_70_ O_15_ |
| 70 | 31.157 | [M+H]^+1^ | Wilforlide A | C_30_ H_46_ O_3_ |
| 71 | 31.54 | [M+H]^+1^ | Roburic acid | C_30_ H_48_ O_2_ |
| 72 | 31.689 | [M+H]^+1^ | 5-Hydroxy-6,7-dimethoxylflavone | C_17_ H_14_ O_5_ |
| 73 | 32.811 | [M+H]^+1^ | Chrysosplenetin B | C_19_ H_18_ O_8_ |
| 74 | 35.931 | [M+H]^+1^ | α-Linolenic acid | C_18_ H_30_ O_2_ |

Table S3

Information of compounds of RTD detected by LC-ESI-MS/MS in different Ion mode.

| **No.** | **RT [min]** | **Ion mode** | **Name** | **Formula** |
| --- | --- | --- | --- | --- |
| 1 | 1.356 | [M-H]^-1^ | 3-[(Carboxycarbonyl)amino]-L-alanine | C_5_ H_8_ N_2_ O_5_ |
| 2 | 1.736 | [M+H]^+1^ | 2-Pyrrolidinecarboxylic acid | C_5_ H_9_ N O_2_ |
| 3 | 1.816 | [M+H]^+1^ | Betaine | C_5_ H_11_ N O_2_ |
| 4 | 1.823 | [M+H]^+1^ | 5-Hydroxymethylfurfural | C_6_ H_6_ O_3_ |
| 5 | 1.904 | [M-H]^-1^ | Citric acid | C_6_ H_8_ O_7_ |
| 6 | 1.96 | [M-H]^-1^ | Mannitol | C_6_ H_14_ O_6_ |
| 7 | 2.471 | [M+H]^+1^ | L-Valine | C_5_ H_11_ N O_2_ |
| 8 | 3 | [M+H]^+1^ | Nicotinic acid | C_6_ H_5_ N O_2_ |
| 9 | 3.048 | [M+H]^+1^ | Guanine | C_5_ H_5_ N_5_ O |
| 10 | 3.456 | [M-H]^-1^ | Fumaric acid | C_4_ H_4_ O_4_ |
| 11 | 4.082 | [M-H]^-1^ | Maleic acid | C_4_ H_4_ O_4_ |
| 12 | 5.467 | [M+H]^+1^ | L-Tyrosine | C_9_ H_11_ N O_3_ |
| 13 | 19.41 | [M-H]^-1^ | Bilobalide | C_15_ H_18_ O_8_ |
| 14 | 20.24 | [M+H]^+1^ | L-Phenylalanine | C_9_ H_11_ N O_2_ |
| 15 | 20.46 | [M-H]^-1^ | p-Hydroxybenzaldehyde | C_7_ H_6_ O_2_ |
| 16 | 20.55 | [M+H]^+1^ | Abscisic acid | C_15_ H_20_ O_4_ |
| 17 | 21.76 | [M+H]^+1^ | p-Coumaric acid | C_9_ H_8_ O_3_ |
| 18 | 21.78 | [M-H]^-1^ | L-Tryptophan | C_11_ H_12_ N_2_ O_2_ |
| 19 | 23.56 | [M-H]^-1^ | Azelaic acid | C_9_ H_16_ O_4_ |
| 20 | 23.92 | [M-H]^-1^ | 4-Hydroxybenzoic acid | C_7_ H_6_ O_3_ |
| 21 | 24.25 | [M-H]^-1^ | Atractyloside A | C_21_ H_36_ O_10_ |
| 22 | 24.28 | [M-H]^-1^ | Baicalin | C_21_ H_18_ O_11_ |
| 23 | 25.04 | [M+H]^+1^ | Palmatine | C_21_ H_21_ N O_4_ |
| 24 | 25.3 | [M+H]^+1^ | Epiberberine | C_20_ H_17_ N O_4_ |
| 25 | 25.98 | [M+H]^+1^ | Calycosin | C_16_ H_12_ O_5_ |
| 26 | 26.37 | [M-H]^-1^ | Dihydrocucurbitacin F | C_30_ H_48_ O_7_ |
| 27 | 26.49 | [M+H]^+1^ | Cinnamic acid | C_9_ H_8_ O_2_ |
| 28 | 27.87 | [M+H]^+1^ | Ganoderic acid G | C_30_ H_44_ O_8_ |
| 29 | 30.08 | [M+H]^+1^ | Formononetin | C_16_ H_12_ O_4_ |
| 30 | 33.44 | [M-H]^-1^ | Cucurbitacin B | C_32_ H_46_ O_8_ |

Table S4

Information of active compounds of AM and RT screened by TCMSP.

| **Mol ID** | **Molecule name** | **OB（%）** | **DL** | **source** |
| --- | --- | --- | --- | --- |
| MOL000033 | (3S,8S,9S,10R,13R,14S,17R)-10,13-dimethyl-17-[(2R,5S)-5-propan-2-yloctan-2-yl]-2,3,4,7,8,9,11,12,14,15,16,17-dodecahydro-1H-cyclopenta[a]phenanthren-3-ol | 36.23 | 0.78 | AM |
| MOL000098 | quercetin | 46.43 | 0.28 | AM |
| MOL000211 | Mairin | 55.38 | 0.78 | AM |
| MOL000239 | Jaranol | 50.83 | 0.29 | AM |
| MOL000296 | hederagenin | 36.91 | 0.75 | AM |
| MOL000354 | isorhamnetin | 49.6 | 0.31 | AM |
| MOL000371 | 3,9-di-O-methylnissolin | 53.74 | 0.48 | AM |
| MOL000374 | 5'-hydroxyiso-muronulatol-2',5'-di-O-glucoside | 41.72 | 0.69 | AM |
| MOL000378 | 7-O-methylisomucronulatol | 74.69 | 0.3 | AM |
| MOL000379 | 9,10-dimethoxypterocarpan-3-O-β-D-glucoside | 36.74 | 0.92 | AM |
| MOL000380 | (6aR,11aR)-9,10-dimethoxy-6a,11a-dihydro-6H-benzofurano[3,2-c]chromen-3-ol | 64.26 | 0.42 | AM |
| MOL000387 | Bifendate | 31.1 | 0.67 | AM |
| MOL000392 | formononetin | 69.67 | 0.21 | AM |
| MOL000398 | isoflavanone | 109.99 | 0.3 | AM |
| MOL000417 | Calycosin | 47.75 | 0.24 | AM |
| MOL000422 | kaempferol | 41.88 | 0.24 | AM |
| MOL000433 | FA | 68.96 | 0.71 | AM |
| MOL000438 | (3R)-3-(2-hydroxy-3,4-dimethoxyphenyl)chroman-7-ol | 67.67 | 0.26 | AM |
| MOL000439 | isomucronulatol-7,2'-di-O-glucosiole | 49.28 | 0.62 | AM |
| MOL000442 | 1,7-Dihydroxy-3,9-dimethoxy pterocarpene | 39.05 | 0.48 | AM |
| MOL004355 | Spinasterol | 42.98 | 0.76 | RT |
| MOL006756 | Schottenol | 37.42 | 0.75 | RT |

Table S5

The original date of Drugs-targets-pathways-disease network.

| **Drugs** | **Target points** | **Signaling pathways** | **Disease** |
| --- | --- | --- | --- |
| AM, RT | PIK3R1, EGFR, CCND1, AKT1, MAPK1, RAC1, HRAS, MAPK3, HSP90AA1, IL2, PTK2, PIK3CA, CDK2 | PI3K-Akt signaling pathway | Melanoma |
| AM, RT | PIK3R1, EGFR, CCND1, AKT1, MAPK1, HRAS, MAPK3, PIK3CA, CDK2 | FoxO signaling pathway | Melanoma |
| AM, RT | SRC, PIK3R1, PTK2, PIK3CA, AKT1, MAPK1, RAC1, HRAS, MAPK3 | VEGF signaling pathway | Melanoma |
| AM, RT | JUN, SRC, PIK3R1, EGFR, PTK2, PIK3CA, ABL1, AKT1, MAPK1, HRAS, MAPK3 | ErbB signaling pathway | Melanoma |
| AM, RT | SRC, PIK3R1, EGFR, AKT1, MAPK1, RAC1, HRAS, MAPK3, PIK3CA | Rap1 signaling pathway | Melanoma |
| AM, RT | PIK3R1, EGFR, ABL1, AKT1, MAPK1, RAC1, HRAS, MAPK3, PTPN11, PIK3CA | Ras signaling pathway | Melanoma |
| AM, RT | HDAC1, SRC, PIK3R1, PIK3CA, CCND1, AKT1, MAPK1, HRAS, MAPK3 | Thyroid hormone signaling pathway | Melanoma |
| AM, RT | PIK3R1, EGFR, PIK3CA, AKT1, MAPK1, MAPK3 | HIF-1 signaling pathway | Melanoma |
| AM, RT | PIK3R1, PIK3CA, AKT1, MAPK1, FYN, RAC1, HRAS, MAPK3 | Fc epsilon RI signaling pathway | Melanoma |
| AM, RT | JUN, PTPN11, PIK3R1, PIK3CA, ABL1, AKT1, MAPK1, RAC1, HRAS, MAPK3 | Neurotrophin signaling pathway | Melanoma |
| AM, RT | SRC, PIK3R1, PIK3CA, CCND1, AKT1, MAPK1, HRAS, MAPK3 | Prolactin signaling pathway | Melanoma |
| AM, RT | JUN, HSP90AA1, SRC, PIK3R1, EGFR, PIK3CA, AKT1, MAPK1, HRAS, MAPK3 | Estrogen signaling pathway | Melanoma |
| AM, RT | JUN, PIK3R1, IL2, PIK3CA, AKT1, MAPK1, FYN, HRAS, MAPK3 | T cell receptor signaling pathway | Melanoma |
| AM, RT | JUN, PIK3R1, PIK3CA, AKT1, MAPK1, MAPK3 | TNF signaling pathway | Melanoma |
| AM, RT | PIK3R1, PIK3CA, AKT1, MAPK1, FYN, RAC1, HRAS, MAPK3 | Sphingolipid signaling pathway | Melanoma |
| AM, RT | JUN, PIK3R1, PIK3CA, AKT1, MAPK1, RAC1, MAPK3 | Toll-like receptor signaling pathway | Melanoma |
| AM, RT | PIK3R1, PIK3CA, AKT1, MAPK1, MAPK3 | mTOR signaling pathway | Melanoma |
| AM, RT | JUN, EGFR, AKT1, MAPK1, RAC1, HRAS, MAPK3 | MAPK signaling pathway | Melanoma |
| AM, RT | CCND1, CDK2, CDK1 | p53 signaling pathway | Melanoma |
| AM, RT | JUN, PIK3R1, PIK3CA, AKT1, MAPK1, RAC1, HRAS, MAPK3 | B cell receptor signaling pathway | Melanoma |
| AM, RT | PIK3R1, PIK3CA, AKT1, MAPK1, HRAS, MAPK3 | Insulin signaling pathway | Melanoma |
| AM, RT | JUN, PIK3R1, PIK3CA, AKT1, MAPK1, RAC1, MAPK3 | cAMP signaling pathway | Melanoma |
| AM, RT | SRC, PIK3R1, PTK2, PIK3CA, AKT1, MAPK1, RAC1, HRAS, MAPK3 | Chemokine signaling pathway | Melanoma |
| AM, RT | JUN, SRC, MAPK1, HRAS, EGFR, MAPK3 | GnRH signaling pathway | Melanoma |
| AM, RT | PIK3R1, PIK3CA, AKT1, MAPK1, HRAS, MAPK3 | signaling pathways regulating pluripotency of stem cells | Melanoma |
| AM, RT | PTPN11, PIK3R1, IL2, PIK3CA, CCND1, AKT1 | Jak-STAT signaling pathway | Melanoma |
| AM, RT | PIK3R1, PIK3CA, CCND1, AKT1 | AMPK signaling pathway | Melanoma |
| AM, RT | JUN, CCND1, SRC, MAPK1, HRAS, EGFR, MAPK3 | Oxytocin signaling pathway | Melanoma |
| AM, RT | HSP90AA1, MAPK1, MAPK3 | NOD-like receptor signaling pathway | Melanoma |
| AM, RT | JUN, CCND1, RAC1 | Wnt signaling pathway | Melanoma |
| AM, RT | MAPK1, MAPK3 | TGF-beta signaling pathway | Melanoma |
| AM, RT | AKT1, PTPN11 | Adipocytokine signaling pathway | Melanoma |
| AM, RT | MAPK1, AKT1, MAPK3 | cGMP-PKG signaling pathway | Melanoma |
| AM, RT | AKT1 | Glucagon signaling pathway | Melanoma |
